# The Role and Function of Mucins and Its Relationship to Inflammatory Bowel Disease

**DOI:** 10.3389/fmed.2022.848344

**Published:** 2022-05-06

**Authors:** Youra Kang, Hyeonjeong Park, Byung-Ho Choe, Ben Kang

**Affiliations:** ^1^Cell and Matrix Research Institute, School of Medicine, Kyungpook National University, Daegu, South Korea; ^2^Department of Pediatrics, School of Medicine, Kyungpook National University, Daegu, South Korea

**Keywords:** mucins, mucus, inflammatory bowel diseases, ulcerative colitis, Crohn's disease

## Abstract

Mucus is present throughout the gastrointestinal tract and is essential for regulating gut microbiota homeostasis and preventing disease by protecting the gastrointestinal barrier from microorganisms, pathogens and toxins or other irritants. Mucin (MUC)-2 is a secreted protein produced by epithelial goblet cells as the main component of mucus. Defects in the gastrointestinal tract, such as inflammation and ulcers, cause damage to the mucus barrier, which can worsen mucus quality and reduce mucus production. Therefore, we would like to review the characteristics of MUC2 and its role in intestinal disorders and highlight the importance of further studies. We also investigated whether the role of MUC2 differs between children and adults, ulcerative colitis (UC) and Crohn's disease (CD).

## Introduction

Inflammatory bowel disease (IBD) is a digestive disorder in which chronic inflammation repeats exacerbation and improvement. The two main types of IBD are ulcerative colitis (UC) and Crohn's disease (CD). IBD is a multifactorial disease, with both genetic and environmental factors contributing to the disease. In particular, loss of intestinal epithelium completeness plays a very important role in IBD (1, 2). Dysfunction of the innate immune system due to abnormal signal transduction caused by pathogens, excessive leakage of bacterial antigens into the mucosa, or inappropriate immune responses due to changes in the composition of the intestinal microflora induce an inflammatory response that progressively degrades the intestinal epithelium. This allows more antigen to leak and worsens inflammation, further damaging the barrier, so the integrity of the mucosal barrier is a key factor in determining the status of IBD (3, 4).

Given the role of the mucus layer, there is growing interest in it because loss of intestinal barrier integrity can cause or exacerbate the progression of inflammatory diseases of the gastrointestinal tract, such as IBD. Although the prevalence is continuously increasing, the cause and treatment are still difficult to identify (5). In particular, IBD onset during childhood and adolescence affects more than 70,000 children in the United States, accounting for 25% of all patients with IBD cases (6–11). About 20–30% of pediatric patients with IBD require surgery within 10 years of diagnosis, so they are more severe than adults, and many receive additional surgical intervention in consideration of recurrence and complications (12–16).

Early diagnosis and treatment of pediatric patients with IBD is very important because children and adolescents are often accompanied by symptoms such as growth retardation and depression due to the characteristics of growth and puberty. In addition, since it requires active treatment from the early stage of diagnosis, high medical costs cannot be ignored. Therefore, to identify differences between adult and pediatric patients with IBD and to enable them to be applied to treatment and prevention, this review highlights the characteristics of MUC2 mucin and its role in IBD, factors affecting MUC2, and differences between IBD in adults and children.

## Structure and Classification of Mucins

**Figure 1:**
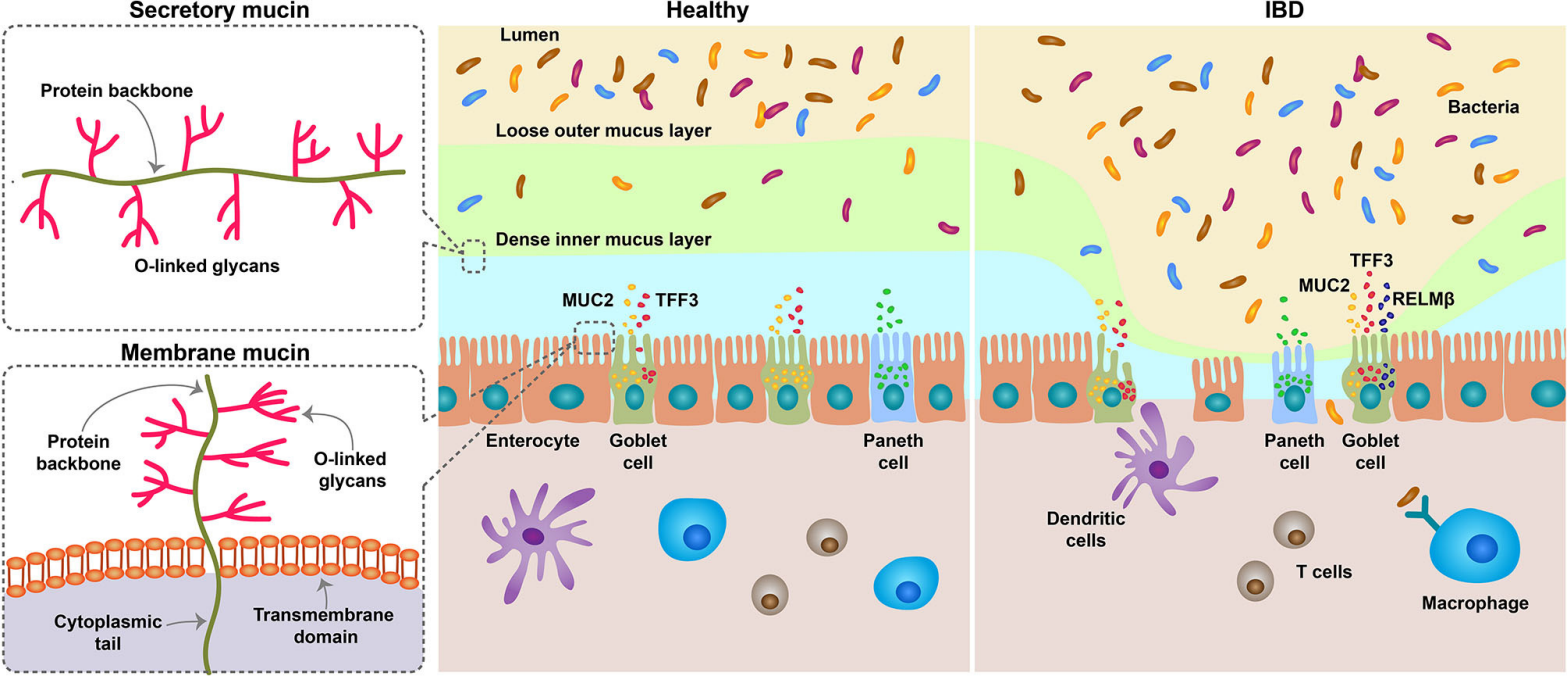
Schematic diagram depicting the role of MUC2 in IBD. The mucous membrane consists of two layers, a loose outer layer, which is permeable to bacteria, and a tightly attached inner layer, which cannot penetrate. Mucins are produced by goblet cells of the intestinal epithelium and are divided into secretory and membranous types. Secreted mucins are gel-forming mucus, composed mostly of MUC2, and provide viscoelasticity. Although the degradation and synthesis of mucin are generally in equilibrium, the number of goblet cells is reduced in the IBD state, and thus the secretion of mucin is also reduced. It also increases the number of immature goblet cells that produce incomplete mucus. When the mucous membrane breaks down, the bacteria in the lumen penetrate inside and cause inflammation. MUC, Mucin; IBD, inflammatory bowel disease.

From MUC1 to MUC22, up to 22 distinct mucin genes have been identified in the sequence of discovery. They are expressed according to tissues and cell types and they are broadly categorized into two types: secretory and membrane related (17–23). MUC2, MUC5AC, MUC5B, and MUC6 are gel-forming secretory mucins found on the mucosal surface. However, MUC1, MUC3, MUC4, MUC13, and MUC17 are membrane-associated mucins found in the apical membranes of epithelial cells (Figure 1). MUC1, MUC2, MUC3, and MUC4 are the predominant mucus detected in colorectum, with MUC2 being produced specifically in goblet cells (21, 24, 25). MUC2 exhibits similar structural and physicochemical properties to other secreted mucins expressed in the gastric and respiratory glandular epithelium, such as MUC5AC, MUC5B, and MUC6. MUC2 monomer contains over 5,000 amino acids and is rich in proline, serine, and threonine (19, 26, 27). MUC2 consists of five distinct regions, including an N-terminal domain comprising von Willebrand D4 domain and CysD domain, a small PTS domain, another CysD domain, a large PTS domain, and a C-terminal part containing von Willebrand D4-C domains, and a cystine knot domain (28).

## MUC2 Biosynthesis, Secretion and Regulation

MUC2 is expressed early in fetal development and is detected in the colon at 12 weeks of gestation and is expressed by individual cells that are goblet cell precursors (29). And most of these cells are localized in the crypt region (30). In the normal colon, MUC2 is expressed in goblet cells (31). Several bioactive factors that enhance mucin secretion regulate mucin synthesis. According to numerous research, mucin expression is regulated by transcriptional or epigenetic regulation (18, 32, 33). MUC2 transcription is regulated by signal transduction pathways, which target transcription factors binding to specific regions of the promoter. Activation of nuclear factor (NF)-κB, a transcription factor, is common during inflammation of the gastrointestinal tract, and the promoter of MUC2 contains an NF-κB binding site. Several pathways activate NF-κB, and the representative pathways are the Ras-mitogen-activated protein kinase (MAPK) pathway through lipopolysaccharide (LPS) and the phosphoinositide 3-kinase (PI3K)/Akt pathway by tumor necrosis factor (TNF)-α. Interleukin (IL)-4 and IL-13 also activate NF-κB through MAPK and upregulate MUC2 transcription (34–36). In contrast, the neuropeptide hormone vasoactive intestinal peptide upregulates MUC2 transcription by activating the transcription factor cAMP response element-binding protein (CREB)/cyclic AMP-dependent transcription factor (ATF)-1, whereas prostaglandin PGE2 also induces MUC2 transcription by activating CREB/ATF1 (37). A recent study reported that the transcription factor SAM pointed a domain-containing Ets (SPDEF) can upregulate MUC2 production by stimulating the differentiation of secretory progenitors into Paneth and goblet cells (38). Epigenetic studies have revealed that MUC2 expression can be downregulated by methylation of CpG islands in specific regions of the MUC2 promoter. Additionally, we reported that MUC2 gene expression is regulated by epigenetic mechanisms of DNA methylation and histone modification of specific regions of the MUC2 promoter (39, 40).

## Intestinal Mucus Layer

Absorption of nutrition is an important function of the small intestine, and the mucous system facilitates this by lubricating and protecting against endogenous enzymes and microorganisms. Mucus entirely covers the surface of the epithelium (20). The intestinal mucus layer consists of a dense mesh-like network of MUC2 secreted by goblet cells, which serve as host defense against endogenous and exogenous irritants and microbial adhesion and invasion but permit the transport of nutrients. The mucus layer is primarily composed of MUC2, but also contains other goblet cell products, such as trefoil factor (TFF)-3, resistin-like molecule (RELM)-β, and Fc fragment of IgG binding protein (Fcgbp) (41–44). The mucous membrane has two mucus layers, which are densely and firmly attached to the interior and loosely attached to the exterior compared to the interior. The thickness of the inner and outer mucus layers was similar in the stomach and jejunum, increased significantly in the ileum, and was thickest in the colon. The thickness of the entire mucus layer exhibited a similar trend. The thickness of the mucus layer is maintained by a balance of synthesis, secretion, and degradation, which is regulated by glycosidases, proteases, and other enzymes (45, 46) (Figure 1).

## Mucus Barrier Alterations in IBD

### MUC2 Alterations

MUC2 plays a significant role in the pathogenesis of IBD, and deficiency of MUC2 mucin can change the composition of the mucus, which can affect the pathogenesis of IBD. UC is a disease of the inner colonic mucous layer, and the host's immune response to the microbiome increases with increased exposure to bacteria.

UC was observed to be thinner and more discontinuous in mucus than CD, and less MUC2 was excreted during the active phase of UC. The decreased secretion of MUC2 may be attributed to the translational or post-translational modifications, and it is also associated with a decrease in goblet cells (47, 48). Moreover, patients with UC have fewer goblet cells and decreased MUC2 synthesis and secretion, especially at the onset of severe disease, enabling direct interaction between the colonic microbiota and the epithelial barrier (49). Patients with UC in the active phase showed low levels of goblet cell differentiation factors HATH1 and kruppel-like factor (KLF)-4 (50). The results of genome-wide association studies (GWAS) showed that IBD pathogenesis was associated with mutations in the MUC genes, including MUC3 and MUC19, and variants of MUC2 have also been detected in IBD cases (49, 51–53).

CD affects all parts of the intestine but is most common in the small intestine. CD, as opposed to UC, is characterized by increased mucus production and discontinuous inflammation. Mucus in the small intestine serves as a diffusion barrier, and mucus secretion occurs primarily in the crypt entrance. Genetic associations are more evident in CD than in UC, involving genes, such as nucleotide binding oligomerization domain containing (NOD)-2, autophagy related 16 like 1 (ATG16L1), immunity related GTPase M (IRGM), and X-box binding protein (XBP)-1 associated with autophagy, unfolded protein responses, and bacterial detection and defense (54–56).

The failure to restrict bacterial access to the epithelium despite increased levels of mucus production in CD patients indicates that mucus level and secondary structural modifications are critical for mucus barrier function. The viscoelastic function of mucus depends on proper glycosylation, sulfation, and sialylation (4, 49). People and mice with IBD have higher levels of sulfide, a product of sulfate-reducing bacteria, which reduces the disulfide bonds between the mucus and breaks down the mucus network (57, 58). Even in the UC cohort, glycosylation and sulfation defects indicated that the mucus was deformed and unable to act effectively as a barrier (57, 59, 60). Because of the GWAS, a mutation in the core 1 beta-3-galactosyltransferase (β3GalT)-specific molecular chaperone (Cosmc), a chaperone of the T-synthase glycosyltransferase responsible for O-glycan synthesis of mucin protein, was associated with IBD (58, 61). Indeed, Muc2 knockout mice developed spontaneous colitis after 5 weeks of age and exhibited increased susceptibility to DSS-induced colitis. Additionally, mice lacking core 1-derived O-glycans lose mucus complexity and exhibit rapid spontaneous colitis (59). Thus, the mucus barrier acts as a means to prevent or limit the contact of epithelial cells with bacteria and antigens, implying that MUC plays an important role in mucus barrier formation and alleviation of colitis such as IBD.

### MUC2 Related Alterations

#### Glycosylation

The glycosylation degree of mucus is essential for its mucosal protective role, and the MUC2 protein core is generally proteolytic resistant because of glycosylation. Bacteria have exoglycosidase, which releases monosaccharide residues and uses them as an energy source. Under normal conditions, the mucus broken down by bacteria is balanced with the new mucus production. However, altered glycosylation can affect this balance, causing glycans to be shortened and digested faster, exposing the protein core and thereby breaking down the mucus barrier faster. Mucus glycosylation defects reduce the mucus layer, placing germs and hosts closer together (59, 62, 63). The glycosylation pattern of MUC2 correlates with the degree of inflammation and disease course, with CD overproduction and abnormal mucin glycosylation, whereas in UC, alterations in MUC2 O-glycosylation in active phase recovered during remission (57, 64).

#### Tight Junction

Since tight junction (TJ) is interdependent with the mucus barrier, the loss of one decreases the other. Mice deficient in Claudin 7, hepatocyte nuclear factor 4 alpha (Hnf4a), and Muc2 develop spontaneous colitis (35, 65, 66). Muc2–/–mice show increased epithelial barrier permeability and mucus defects, and the claudin gene expression is unregulated (66). Also, in Hnf4a–/–mice, the number of goblet cells and mucus decreased (67). This interdependence may result from dysregulation of the signals regulating both mucus and TJ, thereby promoting the inflammatory response that sustains IBD (68).

### Differences Between Adult and Pediatric Patients

We reviewed a number of papers to identify differences in MUC2 expression in UC and CD as well as in adult and pediatric patients. We summarized the MUC and TFF expression characteristics of adult and pediatric IBD patients in Table 1 based on the results of many researchers (Table 1) (11, 24, 69).

**Table 1:** Characteristics of MUC and TFF expression in adults and pediatric IBD patients.

	**Adult**	**Pediatric**
CD	↑Mucus thickness or no change	↓MUC2, TFF2, TFF3 (inflamed TI)
	↓MUC2, MUC3, MUC4, MUC5B, MUC7	↑MUC2, TFF2, TFF3 (non-inflamed TI)>healthy control
	MUC5AC and MUC6 present	↑MUC1, TFF1 (during remission)
	Goblet cell depletion	MUC5AC and TFF1 present
		Goblet cell depletion (TI)
		↓MUC2, TFF2 adjusted for goblet cell density (inflamed)
		↑MUC2, TFF2 adjusted for goblet cell density (non-inflamed)
UC	↓Mucus thickness	TFF1, TFF3, MUC1 not significant
	↓Glycosylation and sulphation	↓TFF2 or not significant (inflamed TI)
	↑Sialylation	↓MUC2 (inflamed TI)
	↓MUC2, MUC9, MUC20	MUC5AC and TFF1 present
	↑MUC1, MUC16	Goblet cell depletion (ascending colon)
	MUC5AC present	
	Goblet cell depletion	

A paper by Bankole et al. (69) published in 2021 systematically reviewed the correlation between adult UC patients and MUC. Among them, according to the contents related to MUC2, when they reviewed the papers reported for 30 years under similar conditions, the decreased and unchanged MUC2 mRNA expression in UC patients were the same in three cases and increased in one case. On the other hand, the protein expression level was increased in five cases, unchanged in two cases, and increased in one case. They explained that the failure to reproduce in all cases was due to differences in mucin expression assessment.

While the correlation between UC and the mucous layer is relatively well-established, it remains controversial in CD. According to the results of the initial study, the thickness of the mucus layer increased in adult CD patients (70), but a recent study showed that it was not significantly different from that of the normal control group (71). However, according to a meta-analysis, total mucin levels were reduced by 34% in adult CD patients (72), and MUC2 expression was observed to be reduced in CD in the ileum similar to UC (73). Grondin et al. (21) suggested that although it is clear that changes in mucin expression and function play a unique role in IBD, characterizing a distinct mucin expression profile is difficult because there is considerable heterogeneity.

Shaoul et al. (30) and Hensel et al. (11), who investigated the expression of MUC and TFF in pediatric IBD patients, showed similar results. In pediatric CD patients with acute inflammation, significantly lower mRNA levels of MUC2, TFF2 and TFF3 were observed in the mucosa of the TI, but higher mRNA levels than healthy controls in tissues without inflammation. Also, MUC1 and TFF1 showed high levels, especially during clinical remission. In UC, the expression level of MUC2 decreased in acute inflammatory conditions, but TFF2 and 3 did not change significantly. In addition, MUC5AC and TFF1, which were not seen in the normal state, were found in the tissues of CD and UC patients, the same as in adults. These results can be seen as a compensatory recovery mechanism for healing and regeneration of mucosal barriers due to tissue damage caused by inflammation.

Alterations in MUC2 amount and quality, and changes in intestinal trefoil factor expression during inflammation, may upregulate MUC5AC and TFF1 expression to compensate for the impaired barrier and repair functions. MUC5AC and TFF1 are not expressed in normal colonic tissue. MUC5AC was sporadically expressed in goblet cells expressing MUC2 in both UC and CD. This pattern was not limited to a specific region of the colon and was not affected by disease severity or local inflammation. It has also been observed in colonic biopsy specimens of autologous restrictive colitis and solitary rectal ulcer syndrome, but unrestricted to IBD. Some of the goblet cells expressing MUC5AC showed expression of TFF1. This change in expression of MUC5AC and TFF1 was suggested to be a non-specific repair function to compensate for the impairment of colonic barrier function (30).

## Conclusions

Mucin acts as an innate host defense mechanism by forming a mucus layer that protects the host from pathogenic microflora invasion and is vital for intestinal microflora formation. Defects in the mucosal barrier result in abnormal bacterial symbiosis and defects in the host's innate and adaptive immune responses, resulting in intestinal inflammatory responses and damage (Figure 1). Therefore, in order to maintain the integrity of the mucosal barrier, the quantity and quality of mucus are also important, and MUC, which is a component of mucus, is inevitably important.

Mucin genes were identified from MUC1 to MUC22 in the order of discovery, and are largely divided into secretory and membrane-related types. Among them, MUC1, 2, 3 and 4 are mucins detected in the colorectal, and MUC2 is the main mucin produced specifically in goblet cells. MUC2 is expressed in colon goblet cells of UC and CD patients as well as healthy individuals. When the colon is depleted of goblet cells due to IBD and other inflammatory conditions, the expression of MUC2 is also reduced and remains in a relatively immature state. This mucus loses its barrier function and exposes the mucous membrane to inflammatory substances. The factors that cause IBD and inflammatory disease are very diverse, but when the integrity of the mucus barrier is lost, it can be a key factor in exacerbating the disease state.

When reviewing the results of various researchers, there are not many studies targeting pediatric IBD patients and many contradictory results have been reported in adult IBD patients, so it is difficult to clearly conclude that the pattern between adults and children is the same. However, at least according to our investigation, it can be suggested that MUC2 expression is decreased and goblet cell decrease is the same in both adults and children in CD and UC in acute inflammatory state. In addition, in the tissues of CD or UC patients, the opposite expression levels may be observed depending on the state of the tissue with or without inflammation or the degree of remission of inflammation, and these results can be considered as a compensatory repair mechanism for mucosal barrier healing and regeneration.

When IBD occurs, pediatric patients need active treatment with biological agents from the beginning, unlike adult patients. As such, pediatric patients have different symptoms or treatment methods from adult patients, but the level of MUC expression is similar. Most of the experimental systems show reproducible results, but for patients, some studies have weak correlations. This is because the factors involved are very diverse and complex. In addition, MUC expression patterns may vary depending on the presence or absence of inflammation and the degree of inflammation in the tissue sample.

Overall, the complexity and heterogeneity of both MUC and TFF makes it difficult to decipher their diverse roles in the pathophysiological process as either a cause or a consequence of IBD. Although many researchers analyzed the results of IBD patient samples, many were excluded when they attempted to draw conclusions by comparing them to each other under similar conditions. Although some studies have documented detailed conditions, many studies have been conducted under relatively broad conditions. In the case of pediatric IBD patients, the number of studies is very limited, regardless of study comparison conditions. This is probably because the patient's age is very young or the sample volume is small, making it very difficult to obtain sufficient samples for the study. To overcome these problems, it is necessary to analyze the sample by subdividing it under more various conditions, and accordingly, it is believed that more consistent results can be derived. In addition, if the criteria for comparative conditions are established and shared, clinical results will be much richer and comparative analysis will be possible for each researcher under the conditions they want. More clinical findings and further studies in pediatric IBD and other inflammatory diseases continue to be needed in the future to determine immune function and potential interaction partners.

## Author Contributions

YK and HP contributed in the acquisition, analysis and interpretation of data, and drafting of the initial manuscript. B-HC contributed in the acquisition, analysis and interpretation of data, and critical revision for important intellectual content. BK contributed in the conception of the study, acquisition, analysis and interpretation of data, and critical revision for important intellectual content. All authors approved the final version of the manuscript and agreed to be accountable for all aspects of the work.

## Funding

This work was supported by the following grants: National Research Foundation of Korea (NRF) grant funded by the Korea Government (MSIT) (No. 2021R1C1C2005429).

## Conflict of Interest

The authors declare that the research was conducted in the absence of any commercial or financial relationships that could be construed as a potential conflict of interest.

## Publisher's Note

All claims expressed in this article are solely those of the authors and do not necessarily represent those of their affiliated organizations, or those of the publisher, the editors and the reviewers. Any product that may be evaluated in this article, or claim that may be made by its manufacturer, is not guaranteed or endorsed by the publisher.
